# 716. Effect of Carbapenem-resistant vs. Carbapenem-susceptible *Enterobacterales* infections on patient outcomes at an academic medical center

**DOI:** 10.1093/ofid/ofad500.778

**Published:** 2023-11-27

**Authors:** Justin A Clark, David Burgess, Daniella Moga

**Affiliations:** University of Kentucky College of Pharmacy, Lexington, Kentucky; UK HealthCare, Lexington, KY; University of Kentucky, Lexington, Kentucky

## Abstract

**Background:**

Carbapenem-resistant *Enterobacterales* (CRE) infections have been shown to lead to excess mortality in patients. To estimate the impact of CRE at an academic medical center not in an endemic area for CRE infections, we sought to compare them against patients having infections by carbapenem-susceptible *Enterobacterales* (CSE).

**Methods:**

This was a retrospective cohort study performed at UK Healthcare Medical Center which assessed admissions between January 1, 2010 – December 31, 2019. The index date was the first positive culture of *E. coli, E. cloacae, K. aerogenes, K. oxytoca,* and/or *K. pneumoniae*. Patients having a CRE culture on index were included in the exposure group, and those having all CSE cultures were included in the comparator group. Exclusion criteria were age < 18 years old, pregnancy, endocarditis, osteomyelitis, necrotizing fasciitis, or cystic fibrosis. Outcomes of interest were 14- and 30-day composite of all-cause mortality or discharge to hospice with follow-up beginning from the index culture. Relative risk (RR) and hazard ratio (HR) were utilized to compare outcomes and inverse probability of treatment weighting (IPTW) was used for statistical adjustment.

**Results:**

Across the decade, 128 patients were included in the CRE group and 6,953 in the CSE group. IPTW-adjusted RR and HR [95% CI] of composite outcome were 1.11 [0.7, 1.74] and 0.98 [0.61, 1.57] after 14 days and 1.19 [0.8, 1.77] and 0.99 [0.65, 1.51] after 30 days, respectively. When utilizing an exposure definition of carbapenem non-susceptible *Enterobacterales* (CNSE), the RR and HR were

1.11 [0.79, 1.57] and 0.95 [0.66, 1.36] after 14 days and 1.25 [0.93, 1.68] and 1.02 [0.75, 1.4] after 30 days. When using the non-susceptible exposure and only including patients with bloodstream infections on index, the RR and HR were 1.50 [0.93, 2.43] and 1.42 [0.85, 2.36] after 14 days and 1.48 [0.96, 2.29] and 1.38 [0.85, 2.24] after 30 days.

**Conclusion:**

Although the composite outcomes between the CRE and CSE groups were not statistically significantly different, an increased risk appeared to exist in follow-up analysis. Continued efforts including observations across multiple institutions are needed to further characterize risks associated with CRE infections.

**Disclosures:**

**All Authors**: No reported disclosures

